# Analysis of community-based studies related with knowledge, awareness, attitude, and behaviors towards HPV and HPV vaccine published in Turkey: A systematic review

**DOI:** 10.4274/jtgga.galenos.2019.2019.0071

**Published:** 2020-06-08

**Authors:** Serpil Özdemir, Rabia Akkaya, Kazım Emre Karaşahin

**Affiliations:** 1 Department of Public Health Nursing, University of Health Sciences Turkey, Gülhane Faculty of Nursing, Ankara, Turkey; 2 Department of Obstetrics and Gynecology, University of Health Sciences Turkey, Gülhane Training and Research Hospital, Ankara, Turkey

**Keywords:** Human papilloma virus, HPV vaccine; knowledge, awareness, attitude, public health

## Abstract

Human papilloma virus (HPV) vaccine is a proven method for preventing HPV-related cancers and genital warts, especially preventing cervical cancer. It is aimed to systematically review and synthesize conclusions in detail from community-based studies published in Turkey between 2009 and 2019, which evaluate the knowledge, awareness, attitude, and behaviors of individuals towards HPV and HPV vaccination. This systematic review is conducted based on the PRISMA reporting method and includes community-based, descriptive cross-sectional and cross-sectional studies published between 2009 and 2019. In this systematic review, 5132 studies from six databases were scanned in total. It was determined that there were 23 studies that met the eligibility criteria for this systematic review. In the reviewed studies, it was determined that the rate of “Hearing of HPV before” was 3.8% at the lowest and 57.0% at the highest, and the rate of “Hearing of HPV vaccine before” was 2.2% at the lowest and 74.7% at the highest. In the reviewed studies, it was reported that although parents’ willingness to have their daughters vaccinated with HPV vaccine varied between 14.4% and 68.0%, their willingness to have their sons vaccinated with HPV vaccine varied between 11.0% and 62.0%. In addition, it was reported that the lowest rate of vaccination with HPV vaccine among participants was 0.3% at the lowest and 6.0% at the highest. Consequently, it is considered that conducting common, systematic, and continuous health education programs aimed at both sexes and including both parents, which will increase the knowledge and awareness on HPV and its vaccine, would provide positive attitudes, and will be effective in protecting against HPV-related cancers.

## Introduction

Human papilloma virus (HPV) infection, which is sexually transmitted to both males and females, is a global epidemic (1,2,3). Approximately 75% of sexually active individuals encounter HPV at some time in their lives (4). Thirteen known carcinogenic types of HPV, which have approximately 200 diagnosed types, may become cancerous by causing chronic and progressive infection (5). HPV-related cancers are listed as cervical, vulvar, vaginal, anal, rectal, penile, and oropharyngeal cancers (5,6). According to data from the surveillance program conducted by the Centers for Disease Control and Prevention in the United States of America between 2008-2012, it is reported that 38,793 people on average were diagnosed as having HPV-related cancer and 59% of whom were females and 41% were males (6). In the last five years in Turkey, the reported prevalences of cervical cancer, vulvar cancer, anal cancer and penile cancer were 16.09%, 1.82%, 1.09%, and 0.16%, respectively (7).

HPV vaccine is a proven method for preventing HPV-related cancers and genital warts, especially preventing cervical cancer (8,9). It is reported that vaccines containing HPV16-18 types prevent 63% of all HPV-related cancers; vaccines containing nine types of HPV (HPV6-11-16-18-31-33-45-52-58) provide protection against cervical, vulvar, vaginal, and anal cancers by 90% (6,10). Throughout the world and in Turkey, HPV vaccines are recommended to individuals from both sexes between the ages of 9 and 26 years and before the first sexual experience (11,12). The HPV vaccine, the safety of which has been verified by the European Medicines Agency (13), is included in national vaccination program in many countries, but it is not included in Turkey’s national vaccination schedule (13,14,15).

## Objectives

The literature reported that negative attitude and behaviors of individuals and parents such as lack of knowledge and low awareness about mode of transmission, protection, and early diagnosis methods of HPV infection, cost of HPV vaccine, potential adverse effects, and suspecting vaccine safety, and negative news on all vaccines prevented the generalization of the HPV vaccination (16,17,18). In the current study, the aim was to systematically review community-based studies that evaluated the knowledge, awareness, attitude, and behaviors of individuals towards HPV and HPV vaccine published in Turkey between 2009 and 2019, and the available conclusions were synthesized in detail.

## Protocol and registration

This systematic review was registered on the International Prospective Register of Systematic Reviews system (approval number: 128435). This systematic review was conducted based on PRISMA reporting method and includes community-based, descriptive cross-sectional and cross-sectional studies published between 2009 and 2019.

## Eligibility criteria

The investigated studies, which were about protection methods against cervical cancer in Turkey published in the last 10 years, were focused on individuals’ knowledge, awareness, attitude, and behaviors on HPV and HPV vaccine. In the literature, no systematical national research report was found on individuals’ knowledge, awareness, attitude, and behaviors on HPV and the HPV vaccine (2). In this systematic review, it was decided that synthesizing community-based studies would be appropriate by anticipating that they would reflect the current status of the community at risk in terms of HPV infection in Turkey. In this respect, eligibility criteria were based on the literature as follows: (1) descriptive cross-sectional and cross-sectional research design published in a national or international peer-reviewed journal; (2) conducted within the borders of the Republic of Turkey; (3) published between 2009 and 2019; (4) sample consisting of healthy/sick individuals. Review articles, letters to the editor, qualitative studies, case-control studies, congress proceedings, and theses were excluded from the systematic review.

## Information sources

Studies included in the systematic review were obtained as a result of comprehensive review of EBSCO, Google Scholar, Proquest, PubMed, Springer, and TR index databases between March 1^st^ and 4^th^, 2019.

## Search

Keywords in English used in the review were “Turkey”, “HPV”, “Human Papilloma Virus”, “HPV vaccine”, “knowledge”, “awareness”, “attitudes”, “behavior”; and “Türkiye”, “İnsan Papilloma Virüsü”, “Human Papilloma Virüsü”, “HPV aşısı”, “bilgi”, “farkındalık”, “tutum” and “davranış” words were used in the Turkish database.

## Study selection

**Figure 1:**
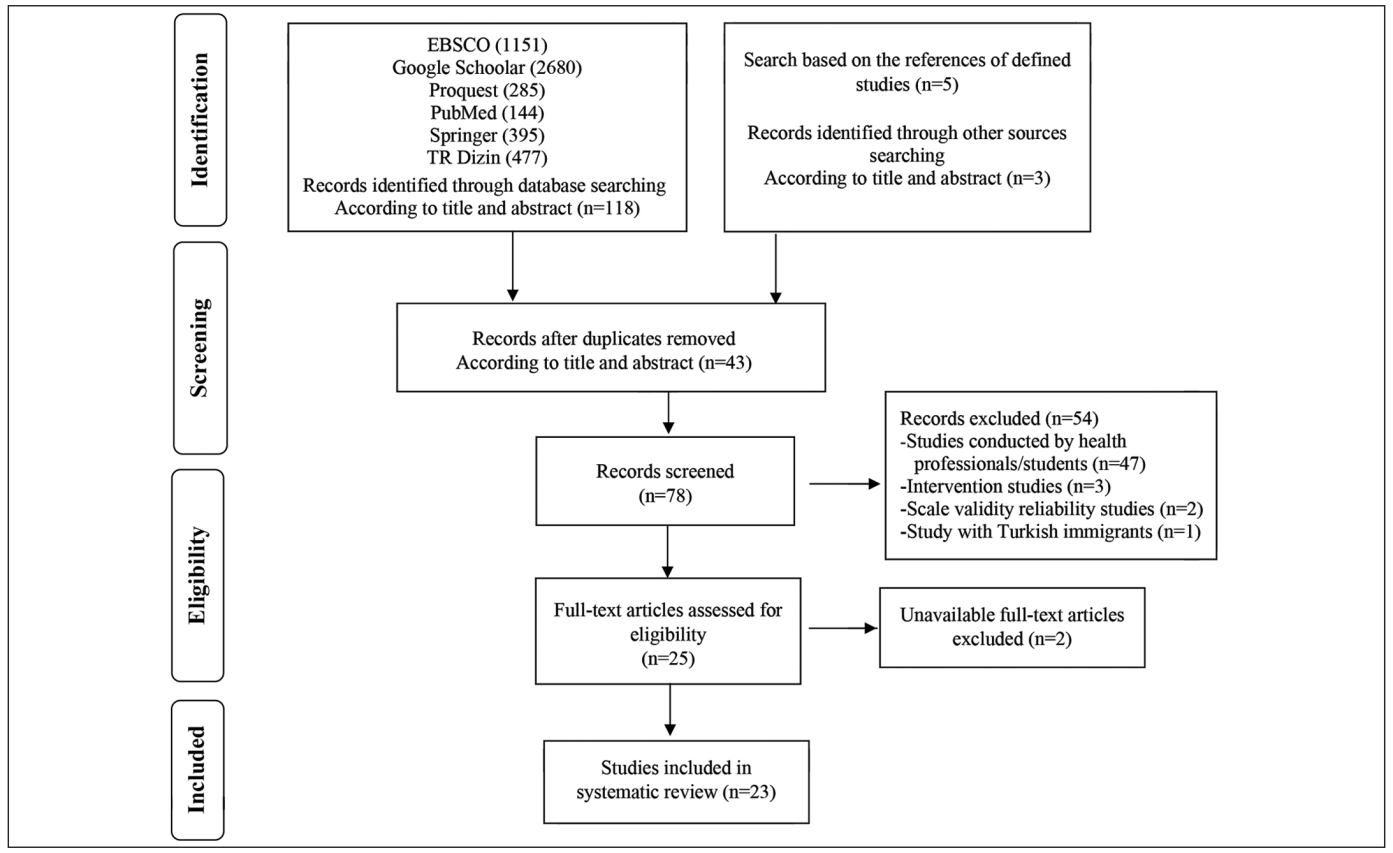
PRISMA flow diagram of the systematic review

In this systematic review, as a result of comprehensive scanning of the databases, 118 research reports were identified according to the titles and abstracts that met the eligibility criteria. It was observed that there were 43 recurring studies among this research. In the assessment according to title and abstract after the recurring researches were identified, studies conducted by healthcare professionals/students (n=47), intervention studies (n=3), scale validity reliability studies (n=2), and studies conducted on immigrant Turks (n=1) were eliminated because they did not fit the purpose of the systematic review. After this stage, the full texts of the studies were reviewed (n=25). Studies for which the full text was not available were excluded from the systematic review (n=2). Following the assessments, it was determined that there were 23 studies that met the eligibility criteria for this systematic review (Figure 1).

## Data collection process

Evidence centers such as the Cochrane Library and Joanna Briggs Institute (JBI) recommend that the studies addressed in systematic reviews are assessed using standardized critical instruments to determine their scientific value and bias risk according to their objectives, design, and method properties (19). It is reported that valid and reliable instruments for determining the reporting quality of cross-sectional studies are limited (20). To assess the reporting quality and properties of the 23 studies included in this systematic review, the 8-question JBI-Critical Appraisal Checklist for Analytical Cross-sectional Studies, which was developed by JBI, was used (21). In the check list, the quality of the studies was assessed in each question as “1=yes”, “2=no”, “3=unclear”, “4=not applicable” (Table 1).

A data collection form developed by the researchers based on the literature was used to collect the data of the scanned studies included in the systematic review. The data collection form includes the author, year, subject, location, sample size and properties, method, main findings, conclusion, and suggestion titles of the study. Researchers reviewed the full texts of 23 studies in detail and recorded in the data collection form under titles independently from each other. Data collection forms of each study were reviewed by all researchers and the data of the systematic review were established.

## Data items

The collected data was merged under the titles of “Author”, “Year”, “Location of the Research”, “Age Range”, “Health Center”, “Number of Participants”, “Properties of Sample”, “Hearing of HPV”, “Hearing of HPV vaccine”, “Vaccination Rate”, “Willingness to Vaccination for Own Self”, “Willingness to Vaccination for Daughter”, “Willingness to Vaccination for Son”, “Barriers of HPV Vaccine”, “Source of HPV Knowledge”, “Willingness to have Education about HPV”, “Factors in relation to HPV and Vaccine Knowledge” and “Suggestions” (Table 2, 3).

## Study selection

In this systematic review, 5132 studies from six databases were scanned in total. It was determined that there were 43 recurring studies out of 118 studies identified according to the titles and abstracts. Number of identified studies was determined as 78. Fifty-four studies that were determined to be beyond the purpose of the systematic review based on the title and abstract were eliminated. Out of the 25 studies whose full texts were to be assessed for eligibility, two studies were excluded because their full texts were not available. The full texts of 23 studies that met the eligibility criteria were included in the scope of the systematic review (Figure 1).

## Reporting characteristics of studies

In the assessment of the studies according to the JBI-Critical Appraisal Checklist for Analytical Cross-sectional Studies, it was determined that eligibility criteria were defined clearly in the sample in 95.6% (n=22) of the studies, and the study subject and methods were explained in detail in 56.5% (n=13) of the studies. It was observed that the researched case was measured in a valid and reliable manner in only 4.3% (n=1) of the studies included in the systematic review, but all of the studies (n=23) used objective criteria for measuring the researched case. It was determined that confounding factors were not identified in 82.6% (n=19) of the studies, and also strategies for coping with confounding factors were not specified. It was determined that the results of all studies (n=23) were assessed using objective criteria and suitable statistical analyses were conducted (Table 1).

## Study characteristics

It was determined that 56.5% (n=13) of the studies included in the systematic review were published between 2009 and 2013 and 56.5% (n=13) were published in international indexed journals. In the studies that were assessed, the age range of the participants varied between 13 and 87 years. The number of participants in the studies was between 229 and 1808. Sixty-five percent (n=15) of the studies addressed only adult women; 21.7% (n=5) addressed adult men and women; 8.6% (n=2) addressed female adolescents and young females; 4.3% (n=1) addressed only males, and 4.3% (n=1) addressed female adolescents and their mothers. In terms of the location of the studies, it was observed that 65.2% (n=15) of the studies were conducted at tertiary healthcare institutions. Studies were conducted in 13 different provinces in total (Table 2).

In the studies, it was determined that the rate of “Hearing of HPV before” was 3.8% at the lowest and 57.0% at the highest, and the rate of “Hearing of HPV vaccine before” was 2.2% at the lowest and 74.7% at the highest. In the assessed studies, it was reported that the parents’ willingness to have their daughters vaccinated with HPV vaccine varied between 14.4% and 68.0%, whereas their willingness to have their sons vaccinated with HPV vaccine varied between 11.0% and 62.0%. In addition, it was reported that the lowest rate of vaccination with HPV vaccine among the participants was 0.3% at the lowest and 6.0% at the highest (Table 2). Two of the reviewed studies investigated the willingness to have education about HPV and its vaccine and it was reported that 69.2% and 95% of the participants were willing to receive health education (Table 3).

In nine studies conducted on in HPV vaccine barriers (43.4%), it was reported that the first three barriers identified were lack of knowledge about HPV and vaccine (40.9% to 76.6%), adverse effects concern (0.9% to 64.5%), and the price of HPV vaccine (0.2% to 49.5%), respectively. According to data obtained from the studies, it was observed that the information source of the participants about HPV and vaccine was healthcare personnel at the rate of 12.3% to 72.2%, and media (e.g. TV, internet, newspapers) at a rate of 23.5% to 88.8% (Table 3). In the studies included in the systematic review, it was reported that awareness, knowledge, and positive attitudes on HPV and vaccine increased as the woman’s/mother’s education level increased in studies that investigated factors in relation to knowledge on HPV and vaccine (60.8%, n=14). In addition, in 17.3% of the studies (n=4), it was reported that awareness, knowledge, and positive attitudes on HPV and vaccine increased in the woman/mother who worked and have high economic level. In this review, it was stated that 82.6% of the studies (n=19) recommended health education, 21.7% (n=5) recommended that more comprehensive and in-depth research should be conducted, and 26.0% (n=6) recommended that policies should be made about vaccine prices and strengthening of primary healthcare services (PHCS) (Table 3).

## Summary of evidence

When the reporting properties of 23 cross-sectional studies included in this systematic review were assessed, it was observed that most of them explained the eligibility criteria in the sample (95.6%) and objective measurements were performed with appropriate statistical analyses and results were reported objectively in all of them. Taking confounding factors under control is quite important in terms of the reliability of the results in cross-sectional studies (22,23). In most of the addressed studies (82.6%), not taking confounding factors under control was considered as a significant limitation in the cross-sectional research design. In addition, using standardized measurement instruments is important in increasing the quality of results obtained in cross-sectional studies (24). In most of the studies reviewed in this study (95.6%), it was determined that standardized valid and reliable measurement instruments were not used. Two valid and reliable scales that assess knowledge, attitude, and beliefs on HPV and vaccine, and which were adapted to Turkish were published in 2016 (25,26). It takes time to publish and announce measurement instruments that are adapted to the culture of a society and use them commonly (27). It was considered that there was a limitation in the studies in terms of using standardized measurement instruments because there were no available standardized measurement instruments until the publication date of valid and reliable HPV and vaccine scales in Turkish, and the measurement instruments were published relatively recently.

In information and awareness studies on HPV and the HPV vaccine that were conducted in developed countries, it was reported that HPV knowledge and awareness were at low-to-moderate levels, and vaccination rates (26%-55%) were not at desired levels, although the willingness for vaccination was high (17,28,29,30,31,32,33,34). In studies conducted in developing countries, it was reported that HPV knowledge and awareness and willingness for vaccination were at low-to-moderate levels, and HPV vaccination rates were quite low (13.3%-16.1%) (35-41). In line with the literature, it is considered that the awareness and knowledge level on HPV and the HPV vaccine (3.8%-57.0%) and willingness for vaccination (6.3%-69.0%) and vaccination rates (0.3%-0.6%) are quite low in this systematic review, which addresses community-based studies in Turkey. It was reported that offering consultancy services on HPV performed by healthcare professionals promoted positive attitudes in these countries by increasing awareness and knowledge on HPV (38,42,43). In Turkey, it is anticipated that the fact that HPV has limited coverage in education programs conducted by healthcare professionals is the cause for the awareness and knowledge on HPV and HPV vaccine and therefore vaccination rates not being at desired levels.

Although HPV immunization willingness was high, various barriers made it difficult to raise vaccination rates to the desired level. HPV vaccination barriers in developed countries were listed as doubts about vaccine safety and efficiency, adverse effect concerns, lack or inconsistency of information about HPV and the HPV vaccine, and the price of the HPV vaccine (29,32,34). In developing countries, HPV vaccine barriers were the lack of awareness of the vaccine, doubting the safety and efficiency of the vaccine, finding it embarrassing to buy the vaccine for sexually transmitted infections, and people thinking that they were not at risk for HPV (35,37,41). In parallel with this, it was determined that the most frequently reported HPV vaccine barriers in studies included in the systematic review were lack of information (40.9% to 76.6%), concerns about the potential adverse effects of the vaccine (0.9% to 64.5%), and the price of the vaccine (0.2% to 49.5%). It was reported that the fact that HPV vaccine was included in the national vaccination schedule in many countries contributed to HPV vaccination in those countries (42). The low HPV vaccination rate obtained in this systematic review could be explained by the fact that HPV vaccine is not included in the national vaccination schedule in Turkey and that vaccine prices are not affordable for the majority of society. In addition, it is anticipated that the fact that healthcare professionals’ level of knowledge and awareness is low, common and continuous health education on HPV is not conducted because of the lack of information about HPV vaccination, therefore resulting in the quite low vaccination levels in Turkey.

The most frequent source of healthcare information in developed countries was healthcare professionals, and media (television, internet, newspaper) at a lower rate (17). In developing countries, it was reported that the most frequent source of healthcare information was media, and healthcare professionals at a lower level (36,38). Similarly, in this review, the most frequently preferred source of healthcare information was the media, and healthcare professionals were preferred at a lower rate. In the studies addressed in this review, it was determined that society was willing and ready at a high rate to obtain information on HPV and the HPV vaccine (69.2%, 95%). Correct and reliable healthcare information should be transferred to society only by specialized healthcare professionals (29,44,45). The misinformation sources and broadcasts against vaccination in the media could prevent society from obtaining correct and reliable information about the HPV vaccine (46). For that reason, it is anticipated that healthcare professionals, who are the reliable sources of information on HPV vaccine, can form positive attitudes and behaviors and encourage the society towards vaccination with continuous and common health educations.

In the literature, it was reported that level of knowledge on HPV vaccine increases as people’s education levels and income levels increase (28,30,35,39,40,47). In parallel with the literature, it was determined that level of knowledge on HPV vaccine increased as the education level and income level increased in the studies addressed in this systematic review.

In that respect, it was suggested that groups with lower education and income levels should be addressed primarily in programs in relation to HPV and the HPV vaccine. Consistent with the literature, it was determined that most of the studies addressed in this review recommended health education for increasing knowledge, awareness, and positive behaviors towards HPV and the HPV vaccine (48). Systematic, common, and continuous health education programs conducted by professionals in accordance with the culture of the society are the most effective method for creating healthy behaviors (48).

The systematic review was limited to the studies published in EBSCO, Google Scholar, Proquest, PubMed, Springer, and TR index databases for which full texts could be accessed. There full text of two studies were not accessible.

## Conclusions

It is considered that conducting common, systematic, and continuous health education programs aimed at both sexes and including both parents, which would increase knowledge and awareness on HPV and the HPV vaccine, and provide positive attitudes, will be effective in protecting people against HPV-related cancers (17,31,33,34,36,38). In addition, it will be an important initiative for the protection of public health that healthcare authorities include HPV vaccines in their immunization programs and that policies encourage acceptance of the vaccine in society in countries where the HPV vaccine is not included in national vaccination schedules. In addition, there is a need for studies with methodologically strong designs that test the methods that will provide positive attitudes towards HPV vaccination in society (30,36,38).

## Figures and Tables

**Table 1:**
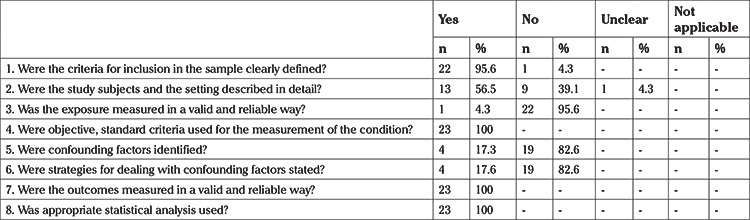
Joanna Briggs Institute-critical appraisal checklist for analytical cross-sectional studies (n=23)

**Table 2:**
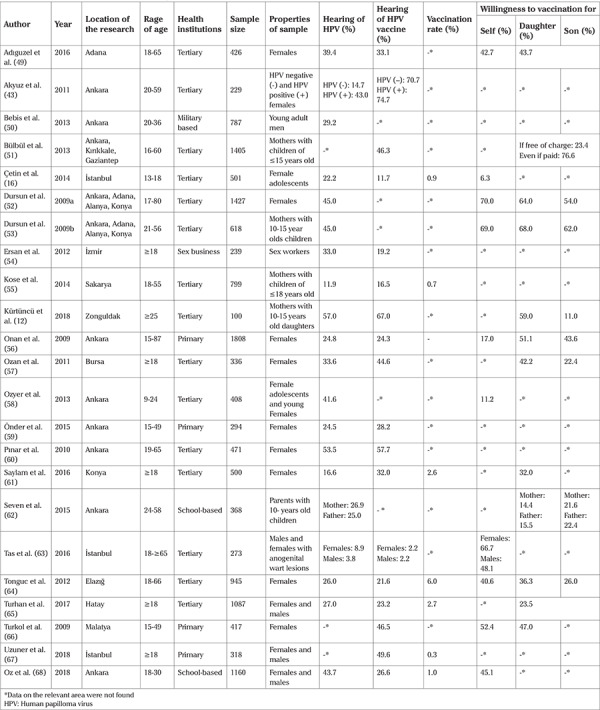
Distribution of the community-based cross-sectional studies (n=23)

**Table 3:**
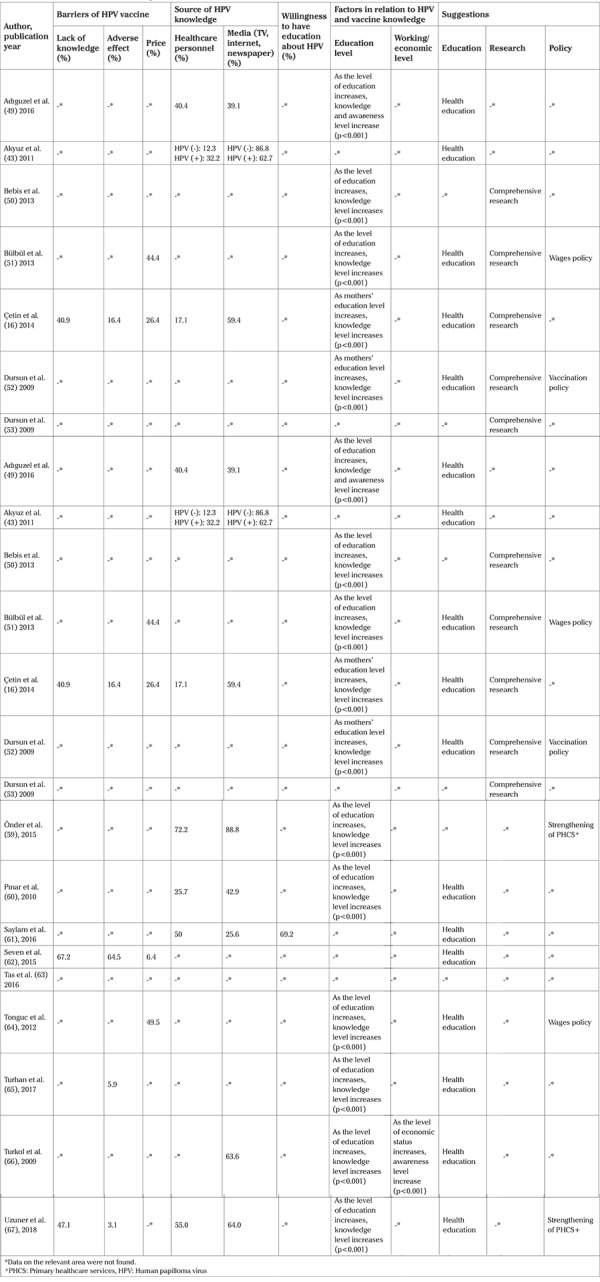
Distribution of community-based cross-sectional studies (n=23)
